# Reply to the letter “Long-COVID may not be explained by skeletal muscle involvement, but rather by other, more compelling pathophysiological concepts”

**DOI:** 10.1055/s-0045-1812889

**Published:** 2025-12-15

**Authors:** João Aris Kouyoumdjian, Leticia Akemi Rama Yamamoto, Carla Renata Graca

**Affiliations:** 1 Faculdade Estadual de Medicina de São José do Rio Preto, Departamento de Ciências Neurológicas, Psiquiatria e Psicologia Médica, Laboratório de Investigação Neuromuscular, São José do Rio Preto SP, Brazil.

Dear Editor,

We thank Finsterer et al. for their interest in our recently published article, “Jitter and muscle fiber conduction velocity in long COVID fatigue” *Kouyoumdjian JA, Yamamoto LAR, Graca CR. Arq Neuropsiquiatr. 2025 Jan;83(1):1–8. doi: 10.1055/s-0045-1802961* .” We appreciate their thoughtful comments and the opportunity to clarify further and discuss some of the points raised.

Our initial impression is that the authors of the letter 1 focused mainly on issues not directly related to our research project, which led to two foundational publications: one presenting reference values for jitter parameters in the tibialis anterior muscle using a concentric needle (CN) and intramuscular micro-axonal electrical activation; 2 and another establishing reference values for muscle fiber conduction velocity (MFCV) in situ, 3 a technique described initially in Erik Stålberg's 1966 Ph.D. thesis 4 and recently revitalized by our team using the single-fiber electromyography (SFEMG) software.

This project ultimately culminated in the current article and a secondary, recently published study, 5 totaling four interdependent publications analyzing jitter and MFCV. We have developed substantial expertise in these methods, as evidenced by key contributions including the multicenter study on CN jitter parameters, 6 the SFEMG guidelines published by the International Federation of Clinical Neurophysiology, 7 and the monograph on jitter using CN electrodes published by the American Association of Neuromuscular and Electrodiagnostic Medicine. 8 More recently, we launched the Atlas of Concentric Needle Jitter, 9 a free online resource with approximately 400 recordingsperformed entirely by Kouyoumdjian and reviewed by Stålberg and Sanders, which also addresses one of the commenters' concerns.

## FIRST ISSUE

None of the cases studied showed signs of inflammatory myopathy or rhabdomyolysis during acute severe acute respiratory syndrome coronavirus 2 (SARS-CoV-2) infection. As previously noted, no patient required hospitalization, aside from brief diagnostic visits. Immune-mediated necrotizing myopathy is rare and typically presents with limb weakness, requiring inpatient care. Mild CK elevations (up to 4-fold) were common, but rhabdomyolysis involves much higher CK levels, dark urine, and renal failure, all of which were absent in our cohort. Thus, there is no evidence of muscle involvement during the acute phase.

## SECOND ISSUE

Myositis-associated antibodies were not assessed, as investigating inflammatory myopathies or their presence during acute infection was beyond the study's scope. In Brazil, such testing was not routinely available during the pandemic. Our focus was on long COVID (LC), which affects an estimated 4 million Brazilians and shares clinical features with myalgic encephalomyelitis/chronic fatigue syndrome (ME/CFS) and postviral fatigue syndrome (PVFS). These conditions lack defined etiopathogenic mechanisms but present overlapping symptoms, including fatigue, sleep disturbances, and brain fog. Diagnosis relies on clinical criteria, including those defined by Fukuda et al. 10 and Carruthers et al., 11 as there are no specific biomarkers.

While jitter studies exist for ME/CFS, no systematic evaluations have been done for LC, nor has MFCV been described in this context. Our study aimed to examine these two peripheral motor parameters—jitter and MFCV—in LC cases, focusing not on rare nosological entities, but on the frequent “muscle-like” symptoms shared across these still poorly defined syndromes: tiredness (relieved by rest), fatigue (not relieved), and postexertional malaise.

## THIRD AND FOURTH ISSUES

The interview and neurophysiological examination were conducted on the same day, with testing following immediately after the interview. In all cases, the interval between acute SARS-CoV-2 infection and the onset of LC symptoms—mostly persistent from the acute phase—exceeded 6 months.

The commenters suggest that other conditions may have developed in the interim to explain possible abnormalities in SFEMG. However, this test evaluates neuromuscular transmission, and disorders in this system are rare, especially given the large number of individuals reporting fatigue. In patients with confirmed SARS-CoV-2 infection and no alternative diagnosis, LC remains a valid clinical entity. Furthermore, we found no statistically significant abnormalities in any group. All results were within normal limits. Slightly increased jitter was observed in one control, one LC-no, and two LC-yes participants, all of whom were negative for anti-acetylcholine (anti-AChR) antibodies and did not exhibit clinical weakness.

## FIFTH ISSUE

During the pandemic, clusters of SARS-CoV-2 infection frequently occurred within families. In a few early cases (12.5%), diagnosis was based on positive antibodies, accepted due to the simultaneous occurrence of illness in multiple relatives. In the majority (87.5%), infection was confirmed by polymerase chain reaction (PCR).

## SIXTH ISSUE

The sixth point suggests we overlooked immunological complications beyond myopathy or neuromuscular transmission disorders in LC. However, our study specifically aimed to assess neuromuscular transmission via SFEMG and MFCV. As noted, LC shares features with ME/CFS and PVFS, conditions with unclear pathophysiology.

Our findings do not support a role for neuromuscular transmission dysfunction or myopathy in the symptoms of LC. While other mechanisms may contribute, exploring them was outside the scope of our study. Notably, the commenters' title “Long COVID may not be explained by skeletal muscle involvement, but rather by other, more compelling pathophysiological concepts” aligns with our own conclusion: “The electrophysiological findings did not indicate that muscle fiber or NMJ dysfunction was a relevant factor in this condition.”

In conclusion, the commenters that the study has limitations affecting its results and interpretation. However, these limitations—primarily the small sample size due to the complexity of the tests—are already acknowledged in our Discussion. In fact, their conclusion aligns with ours: that skeletal muscle involvement alone does not explain LC symptoms, and other pathophysiological mechanisms are likely involved.
